# C-Peptide: A New Mediator of Atherosclerosis in Diabetes

**DOI:** 10.1155/2012/858692

**Published:** 2012-03-22

**Authors:** Dusica Vasic, Daniel Walcher

**Affiliations:** Department of Internal Medicine II-Cardiology, University of Ulm, Albert-Einstein-Allee 11, 89081 Ulm, Germany

## Abstract

Diabetes type 2 and insulin resistance are the risk factors for cardiovascular disease. It is already known that atherosclerosis is an inflammatory disease, and a lot of different factors are involved in its onset. C-peptide is a cleavage product of proinsulin, an active substance with a number of effects within different complications of diabetes. In this paper we discuss the role of C-peptide and its effects in the development of atherosclerosis in type 2 diabetic patients.

## 1. Introduction

C-peptide is a small 31-amino acid peptide, and it is cleaved from proinsulin in the synthesis of insulin [1]. Proinsulin consists of A and B chain and connecting peptide in the middle, called C-peptide. Cleavage of proinsulin takes place in endoplasmatic reticulum of beta pancreatic cells. In addition, C-peptide is stored in Golgi secretory granules and is cosecreted in equimolar amounts into the blood stream together with insulin in response to the glucose stimulation [1, 2]. Amino acid sequences of C-peptide are in different species relatively variable. Nevertheless, C-peptide has several conserved sequences, for example, N terminal acidic region, glycine-rich central segment, and C-terminal pentapeptide [3]. Despite the first reports describing C-peptide as a peptide with little or no biological activity, recent data reports binding of radioactive labelled C-peptide on the cell membranes [4]. Other studies show binding effects stimulating Na-K-ATPase. C-terminal pentapeptide gives full replacement of the entire molecule, which is similar to other peptides with hormone function like gastrin and cholecystokinin [5, 6]. The receptor stays unknown but there is a lot of data demonstrating C-peptide biological effects by activating different signalling pathways, for example binding to pertussis-toxin-sensitive G_i_-coupled receptor on Swiss 3T3 fibroblasts [7, 8] or activating p38 protein kinase pathway in mouse lung capillary endothelial cells [9]. The approach of Luppi et al. detected C-peptide in early endosomes which can be signalling station in the cell, though C-peptide might achieve its cellular effects [10].

There is a certain controversy regarding reported effects of the C-peptide. Its beneficial effects have been demonstrated in long-term complication in type 1 diabetes. Substitution of C-peptide in type 1 diabetes improves glomerular hyperfiltration, hypertrophy, and proteinuria [11–14]. In contrast to this, C-peptide in type 2 diabetes shows proinflammatory and proatherogenic effects [15, 16]. The aim of this paper focuses on the proinflammatory effects of C-peptide and its potential importance in atherosclerosis in diabetic subjects. 

## 2. Atherosclerosis Is an Inflammatory Disease

**Figure 1:**
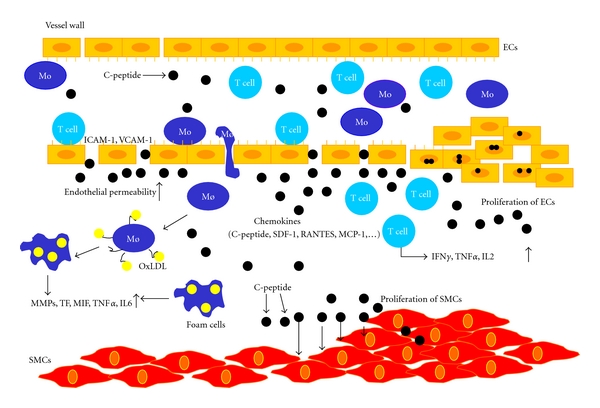
In insulin resistance and early type 2 diabetes insulin levels are increased in circulation. C-peptide levels in blood are increased in equimolar concentration with insulin. C-peptide deposits in subendothelial place in the vessel wall. Deposition is followed by chemotactic effect of C-peptide on the inflammatory cells. It induces migration of monocyte/macrophages and T lymphocytes into the vessel wall. C-peptide has also an effect on the proliferation of smooth muscle cells from the media.

Atherosclerotic lesions are molecular and cellular responses in the vessel wall that have been described as inflammatory disease [17]. Endothelial dysfunction is an early event in atherosclerosis and an important feature of glucose intolerance, diabetes, obesity, and dyslipidemia, as well as a major component of cardiovascular disorders, including hypertension and atherosclerosic diseases [18]. The atherosclerotic plaque consists of necrotic core, calcified regions, foam cells with accumulated lipids, inflamed smooth muscle cells, endothelial cells, lymphocytes, and leukocytes [17]. Minimally oxidised LDL in blood can release bioactive phospholipids that can activate vascular endothelial cells to express leukocyte adhesion molecules, such as vascular cell adhesion molecule-1 (VCAM-1) or intercellular adhesion molecule-1 (ICAM-1) [19]. Highly oxidised LDL can be recognised by monocytes scavenger receptor to be transformed into foam cells (Figure 1). Minimally oxidised LDL-induced expression of adhesion molecules induces initial step in atherosclerosis, leukocyte recruitment, and rolling on the endothelium. Moreover, activated endothelium expresses selectins, monocyte chemoattractant protein-1 (MCP-1), RANTES, and fractalkine, which allow leukocyte adherence to the endothelium [20]. Chemokines are small proteins, and their primary function is activation of specific pertusis-toxin sensitive G-protein-coupled receptors, which results in migration of inflammatory cells [21]. Monocytes and T lymphocytes are migrating into the intima of the vessel wall. Monocytes are expressing scavenger receptor and toll-like receptor, which mediate differentiation into foam cells. These cells in addition play central role in atherosclerotic plaque formation [22, 23]. In atheroma activated macrophages release IL-6, TNF*α*, MIF, and other proinflammatory cytokines and chemokines as well as nitric oxide. This proinflammatory response promotes replication of smooth muscle cells from the media and formation of extracellular matrix [17].

T lymphocytes are entering the subendothelial space as naïve T0 cells. Family of T cell chemoattractants such as IP-10 can in the same way regulate lymphocyte recruitment into the atheroma [24]. Smooth muscle cells are producing extracellular matrix within the vessel wall and in response to atherogenic stimuli they can modify the type of matrix produced. Further, the type of matrix affects the lipid content of the plaque and the proliferative index of the cells attached to the plaque [25]. T lymphocytes release interferon-*γ* (IFN-*γ*) into the plaque, which might block the collagen synthesis in SMC and decrease their ability to renew the collagen. Degradation of extracellular matrix allows penetration of SMCs through elastic laminae and enables plaque to grow [26]. Activated macrophages secrete proteolytic enzymes and matrix metalloproteinases, and that can lead to degradation of the matrix complex of the plaque and destabilisation of the atheroma with increased risk for plaque rupture and can lead to acute clinical events such as myocardial infarction and stroke [27].

### 2.1. Proinflammatory* In Vitro *Effects of C-Peptide

Individuals with diabetes have increased risk of coronary heart disease compared with nondiabetic individuals, and the risk of cardiovascular deaths is as high as in nondiabetic individuals with previous myocardial infarction [28].

Marx et al. reported deposition of C-peptide in the subendothelial space in carotid artery in diabetic subjects [15]. In some of the subjects, deposition of C-peptide was found in the media of the artery. In contrast to this, in nondiabetic patients deposition of C-peptide has not been found. All the 21 subjects involved in the study had deposition of C-peptide, with 77% of them also having infiltration of monocytes and just 57% infiltration of T lymphocytes [15]. Marx and colleagues used these results to propose the hypothesis that C-peptide may have chemotactic effects on the inflammatory cells and might have a role in atherosclerosis (Figure 1). *In vitro* migration assays performed in modified Boyden chambers reported that C-peptide induces migration of T lymphocytes and monocytes/macrophages in a concentration-dependent manner. These effects were similar to those induced with monocyte chemokine MCP-1 or T-lymphocyte chemokine RANTES [15, 29]. In addition, checkerboard analysis in the same study showed that C-peptide induces chemotaxis rather than chemokinesis [29]. Also there are no migratory effects of C-peptide on B cells or neutrophils [30]. C-peptide stimulates specific intracellular signalling pathways in different cell types [8, 9, 31], for example, Na+/K+ATP-ase [5, 32] ERK kinase, PI-3 Kinase [8, 29, 32, 33], and AKT [8, 32, 33]. In T lymphocyte or in monocytes, C-peptide mediates its chemotactic activity through an as-of-yet- unidentified pertussis toxin sensitive G-protein-coupled receptor with subsequent downstream activation of PI3-kinase *γ*. Our experiments demonstrated that a specific inhibitor of Src-kinase, PP2, in addition to transfection of Src siRNA, abolished C-peptide-induced T lymphocyte migration, suggesting that C-peptide also signals through this pathway. Besides, experiments showed that PI-3 kinase activation leads to the involvement of small Rho-GTPases, like RhoA, Rac-1, and Cdc42 in these cells. Furthermore in CD4-positive lymphocytes, C-peptide stimulates phosphorylation of PAK (p21-activated kinase), LIMK (LIM domain-containing protein kinase), and cofilin downstream of Rac-1 and Cdc42, leading to cofilin inactivation and actin filament stabilization. Alternatively, C-peptide activates ROCK (Rho kinase) and MLC (myosin light chain) phosphorylation downstream of RhoA, thereby stimulating myosin-mediated cell contraction [30]. These data supported an active role of C-peptide in chemotaxis of inflammatory cells.

C-peptide has an effect on increased microvascular blood flow in patients with type 1 diabetes [34]. Some studies suggest direct role of endogenous insulin and C-peptide in amelioration of endothelial dysfunction [35]. Additionally, C-peptide increases nitric oxide (NO) production through ERK-dependent upregulation of endothelial nitric oxide synthase (eNOS) gene transcription [36].

In addition, C-peptide positively influences the expression of CD34 scavenger receptor in human THP-1 monocytes. These data suggest that C-peptide may also promote the differentiation of monocyte/macrophages towards foam cells, thus representing another potential proatherogenic effect of C-peptide [37].

Further effects of C-peptide have been investigated on the smooth muscle cells, which are important for the development of atherosclerosis. Stimulation with C-peptide induced proliferation of smooth muscle cells in concentration-dependent manner. Walcher and colleagues showed significantly higher production of KI-67 nuclear protein and 3[H]-thymidine incorporation in vascular cells stimulated with C-peptide. This proliferation was similar to those induced by platelet-derived growth factor (PDGF) [33]. Additionally, C-peptide stimulation induces phosphorylation of protein tyrosine kinase Src and PI-3 kinase, which leads to downstream stimulation of MAP ERK1/2 [33]. It is already demonstrated that activation of ERK1/2 is a crucial step in cell proliferation and differentiation [38]. The downstream control of VSMC proliferation by extracellular stimuli takes place in mid- to late G_1_ phase of the cell cycle, where D-type cyclins promote G_1_- to S-phase transition by leading to Rb phosphorylation [39, 40]. Our data showed an increase in cyclin D1 expression, whereas Rb phosphorylation suggested that C-peptide acts via similar signalling pathways [33].

C-peptide mitogenic effects have been detected in other cell types, like endothelial cells, HEK293 cells, and chondrocytes. When endothelial cells were exposed to C-peptide, a significant increase in cell number of 40% was observed [41] (Figure 1). Another group has found that C-peptide stimulates rRNA synthesis, suggesting that the peptide can have proliferative effects and induces expression of 47S in HCS-2/8 chondrocytes derived from a human chondrosarcoma. After 72 hours of exposure to C-peptide, cell counting under a phase contrast microscope and measurement with a cell proliferation kit and BrdUrd staining established that C-peptide exerts proliferative effects on chondrocytes [42].

## 3. Proinflammatory* In Vivo *Effects of C-Peptide

**Figure 2:**
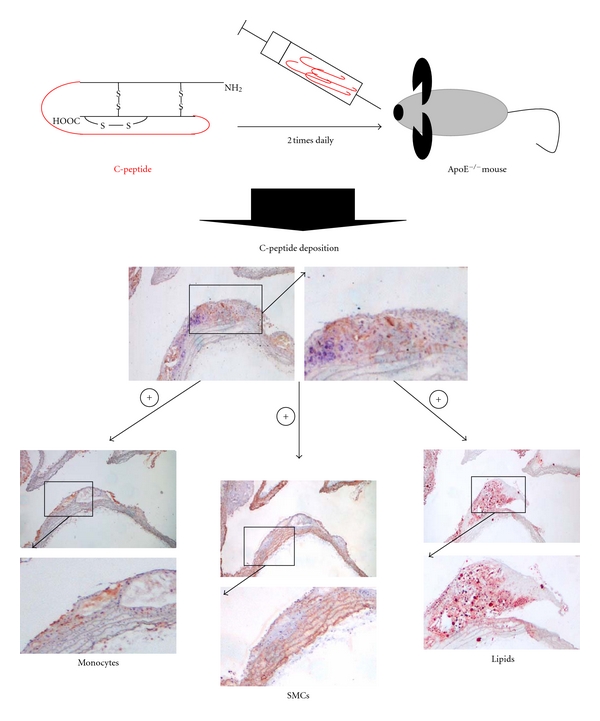
Increased levels of C-peptide in ApoE-deficient mice were established by subcutaneously injections of C-peptide. C-peptide was administrated two times daily by subcutaneous injections for 12 weeks. Deposition of C-peptide in aortic arch has been investigated in mice treated with C-peptide and control mice. Increased deposition of C-peptide in treated mice leads to increased infiltration of inflammatory cells (monocytes/macrophages), increased proliferation of smooth muscle cells from the media, and increased deposition of lipids in aortic arch assessed by immunohistochemistry.

Observational data from previous studies showed deposition of C-peptide in intima of the carotid artery in diabetic individuals [15]. Further, these data showed that C-peptide induces chemotaxis of inflammatory cells *in vitro* and activation of intracellular signalling pathways [29, 30]. These observations needed *in vivo* experimental model to explore effects of C-peptide in onset of atherosclerosis. To test this hypothesis we applied ApoE-deficient mouse model. The animals were divided into two groups. C-peptide group numbered 18, and placebo 17 mice per group [16]. After subcutaneous injections (200 nmol/injection) of dissolved peptide we identify that C-peptide levels in blood increased 4- to 5-fold compared to basal levels (12.9 ± 1.8 nmol/L compared with 2.7 ± 0.8 nmol/L; C-peptide versus placebo; *P* < 0.05). Simultaneously mice were put on the western type diet to trigger atherosclerosis. Immunohistochemical analysis of the aortic arch showed deposition of C-peptide in the early atherosclerotic plaques. Computer-assisted image quantification revealed significantly higher deposition of C-peptide in treated mice, compared to placebo one (2.1 ± 0.4 versus 0.8 ± 0.1% positive area; *P* < 0.01) treated with water (Figure 2). Similar results were obtained in the aortic root (data not shown). After 12 weeks of C-peptide or water sc injections body weight and lipids (total cholesterol, triglyceride, high density lipoprotein, and low density lipoprotein) did not differ between the two treated groups. In addition, glucose and insulin levels showed no differences between groups.

Deposition of C-peptide was followed with increased infiltration of inflammatory cells such as monocytes/macrophages in the aortic arch. Moreover, higher deposition of inflammatory cells has been detected in the aortic root (data not shown). Colocalization of C-peptide with inflammatory cells was already demonstrated in early atherosclerotic plaques of diabetic patients [15]. In contrast to this it has been revealed that C-peptide demonstrates antithrombotic effects *in vivo*. Administration of C-peptide in high doses caused delay in arteriolar and venular thrombus growth in normal and diabetic mice [43].

We already know that diabetes accelerates smooth muscle cell proliferation in atherosclerotic lesions and that it correlates with insulin levels [44]. In a study by Walcher et al., authors revealed that the C-peptide acts as a mitogen on the human and rat arterial vascular smooth muscle cells *in-vitro * [33]. Staining for *α*-actin (Figure 2) in animal model has shown significantly higher content of smooth muscle cells in C-peptide-treated group (C-peptide versus placebo: 4.8 ± 0.6 versus 2.4 ± 0.7% positive area; *P* < 0.01) as well as a trend towards more KI-67 proliferated cells in C-peptide-treated group.

C-peptide had significantly higher deposition of lipids in aortic arch compared with placebo. Lipid deposition in *en face* preparations of the abdominal and thoracic aorta in C-peptide-treated mice did not reach statistical significance compared to placebo mice (C-peptide versus placebo: 5.64 ± 0.69% versus 3.98 ± 0.5%; *P* = 0.07) [16]. A possible explanation could be that C-peptide proinflammatory effects obtained in the ApoE-deficient animals were on top of a high-cholesterol diet. Initial aim of this study was to detect deposition of C-peptide in the vessel wall in an animal model without distinguishing metabolic effects. In the future it would be interesting to use a model of diabetes and atherosclerosis-prone mice fed with high-cholesterol diet such as ob/ob or LDL−/− mice. Furthermore, nothing is known about C-peptide effects on plaque vulnerability or production of metalloproteinases. Future studies should answer these questions.

## 4. Discussion

C-peptide is by now identified as a biologically active substance. Many studies initiate C-peptide as an active peptide hormone with important physiological effects, which affects renal, neuronal, and microvascular functions in patients with diabetes [45–48]. C-peptide increases capillary blood flow in type 1 diabetic patients [49], through increased influx of Ca^2+^ into endothelial cells, which facilitate release of NO from the endothelium. Many studies have demonstrated beneficial effects of C-peptide on the long-term complications in type 1 diabetic patients. This could have an important therapeutic implication [11, 12]. For example, decreased blood flow in the extremities might be prevented by C-peptide [50]. Moreover, improvements of endoneurial blood flow and axonal swelling have been also demonstrated by introduction of C-peptide [51]. In numerous studies of type 1 diabetes glomerular hyperfiltration, hypertrophy, and proteinuria have been reduced by C-peptide [13, 14, 52]. C-peptide treatment improves sensory nerve function in early stage of type 1 diabetic neuropathy [47]. The effects of C-peptide on type 2 diabetes as well as on the cell proliferation and apoptosis are very controversial at present. Levels of inflammation in type 1 and type 2 diabetes are still unknown, but it has been found that plasma levels of IL-6 correlate with C-peptide levels and insulin sensitivity [53]. The metabolic syndrome, prediabetes, and type 2 diabetes mellitus accelerate vascular disease and increase development of the disease [54]. At the moment the reasons for the increased predisposition and progression of atherosclerosis in patients with diabetes are unknown. *In vivo* model from Vasic et al.[16] showed increased deposition of C-peptide in early atherosclerotic lesions in ApoE-deficient mice. C-peptide deposition was followed by recruitment of inflammatory cells into the vessel wall and increased infiltration of monocytes/macrophages as well as increased proliferation of smooth muscle cells. These results are also in agreement with *in vitro* data of Swiss 3T3 fibroblasts, where C-peptide has been shown to activate PI-3 kinase [8] as well as increased expression of PPAR-*γ* regulated CD36 scavenger receptor in human THP-1 monocytes by C-peptide. These results recommend that C-peptide in addition to these effects might promote the differentiation of monocyte/macrophages into foam cells [37]. Our study showed no differences in E-selectin and ICAM-1 levels as well as levels of the inflammatory markers such as TNF*α* and soluble IL-6. An explanation could be that C-peptide was used in this model on top of the hypercholesterinemic diet. But these data are in contrast to several findings in which C-peptide has anti-inflammatory effects and reduced upregulation of cell adhesion molecules under inflammatory conditions [55, 56]. In mice with endotoxic shock, C-peptide treatment improved survival rate and reduced plasma levels of tumour necrosis factor-alpha (TNF*α*), macrophage inflammatory protein-1 alpha, and monocyte chemoattractant protein-1 [57].

We already know that the smooth muscle cells and their secreted products are the main components of advanced atherosclerotic lesions [58]. C-peptide deposition was also found in the media in diabetic patients. Moreover, C-peptide induced proliferation of smooth muscle cells *in vitro*, therefore potentially promoting both the development of atherosclerotic lesions and neointima formation after coronary intervention [33]. After vascular injury, smooth muscle cells start to proliferate and migrate into the developing neointima. They develop into the major cellular substrate of the restenotic tissue [59]. *In vivo* studies showed that downregulation of ERK1/2 inhibits early smooth muscle cell proliferation and neointimal thickening in response to arterial injury [60]. In smooth muscle cells C-peptide induces ERK1/2 signalling. Data obtained from ApoE-deficient mice demonstrated significantly higher content of smooth muscle cells in mouse aortic arch, which was followed with higher deposition of lipids in early atherosclerotic lesions in mice treated with high concentrations of C-peptide [16]. In contrast to this, *in vitro *results by Kobayashi revealed that human C-peptide at high concentrations (100 nmol) suppresses the growth of rat SMCs [61]. A recent study by Cifarelli demonstrated that C-peptide significantly decreases caspase-3 activity and upregulated production of the antiapoptotic factor B-cell CLL/lymphoma 2 (BCL-2) [62]. Anti-inflammatory effects of C-peptide were observed in the study by Chima et al., where C-peptide has been shown to react as inhibitor of lung inflammation following hemorrhagic shock [63].

Conflicting data could be possibly explained with the existence of different circulating insulin and C-peptide levels in diabetes type 2 and diabetes type 1. Most of the studies suggesting anti-inflammatory and antiapoptotic effects of C-peptide performed their experiments on the diverse cell types simulating type 1 diabetes with high glucose levels and low levels of C-peptide where its substitution was beneficial. In regard to this, C-peptide protects endothelial cells from apoptosis and inflammation triggered by high glucose conditions [62]. The situation can be totally different in patients with insulin resistance and type 2 diabetes where high levels of C-peptide could have opposite effects.

A recent study suggests that basal C-peptide levels in type 2 diabetes related to metabolic syndrome correlate to intima-media thickness and C-peptide could be used as surrogate marker of subclinical atherosclerosis [64]. Moreover, Lindahl and colleagues showed that C-peptide stimulates the proliferation of chondrocytes and HEK-293 cells. This regulation of ribosomal RNA means that C-peptide has growth factor activity [42].

In the last few decades C-peptide is presented as an active peptide with diverse effects. Different effects in type 1 and type 2 diabetes seem to be tissue and cell specific. Further work is needed to identify C-peptide receptor and elucidate mechanisms by which C-peptide modulates cell signalling in different cell types.
